# Concrete consequences: construction on prime honey bee habitat doubles foraging distances

**DOI:** 10.1242/bio.061807

**Published:** 2025-05-19

**Authors:** Robert B. J. Ostrom, Margaret J. Couvillon, Bradley D. Ohlinger, Roger Schürch

**Affiliations:** ^1^ Department of Entomology, Virginia Tech, Blacksburg, VA, 24061, USA; ^2^ Odum School of Ecology, University of Georgia, Athens, GA, 30602, USA

**Keywords:** Behavioral analysis, Economic foraging theory, Habitat fragmentation

## Abstract

Human-induced land-use change is a well-documented driver of species decline, including bees, but its true cost may be underestimated. The effects of habitat conversion on honey bee foraging metabolic costs are not well documented. Here, we quantify the impact of land use change on the foraging of freely flying honey bees (*Apis mellifera*) before (2018-2019, *n*=382) and after (2022, *n*=502) their historical foraging habitat is developed. We decoded and analyzed honey bee waggle dances, through which returning foragers communicate the vector of forage. We found that bees increased (from 2.4% to 8.4%) their use of undisturbed microhabitat within the development. The small-scale developments, covering just 1% of the foraging range, nearly doubled flight distance and energy expenditure. Average distance increased from 0.69 to 1.28 kilometers (from 7 to 13 Joules). Our study updates our understanding of land development costs on local bees, revealing concrete consequences to changing land upon which pollinators depend.

## Teaser

**Summary:** Construction on 1% of honey bee foraging range nearly doubles foraging distance and energy expenditure, revealed by waggle dance analysis.

## INTRODUCTION

Habitat loss and degradation, largely caused or accelerated by human activities, is a pernicious driver in the decline of the abundance and diversity of species, a loss so severe that it has been labelled the Sixth Great Mass Extinction (Jaureguiberry et al., 2022). Affected taxa groups include insects, and bees in particular (Winfree et al., 2009; Dicks et al., 2021; Potts et al., 2010), which is alarming because they provide ecosystem services in natural and agricultural settings (Klein et al., 2018). Although previous investigations have established strong correlations between land use changes and bee declines (Winfree et al., 2009; Dicks et al., 2021; Potts et al., 2010), these studies may be underestimating the overall cost. For example, little work has been done, outside of theoretical models, quantifying the real-time energetic cost of land use changes on foraging honey bees if they now must make use of developed areas.

Honey bees (*Apis mellifera*) are a tractable study organism that forage at long distances (von Frisch, 1967; Seeley, 1995) and, importantly, exhibit the waggle dance, a unique behavior where a returning forager communicates the distance and direction to a good resource (von Frisch, 1967), usually nectar or pollen. Because we can observe and analyze these communications in aggregate to determine where the landscape is good for forage, honey bees may serve as bioindicators of habitat quality for themselves and other bees (Couvillon et al., 2014a). Additionally, honey bees are one of the most economically impactful pollinators (Southwick and Southwick, 1992; Klein et al., 2006), with documented declines in North America (Goulson et al., 2015; Pettis and Delaplane, 2010; Aizen et al., 2009) driven, in part, by a lack of abundant, diverse forage in the landscape that leads to nutritional stress (Smart et al., 2016, 2017). Previously it was shown that landscape composition is important for individual and population level honey bee health (Smart et al., 2016, 2017; Lecocq et al., 2015; Otto et al., 2016; Quinlan et al., 2021). However, what is not known is how land use composition changes might impact bees, especially regarding their foraging.

**Fig. 1.**
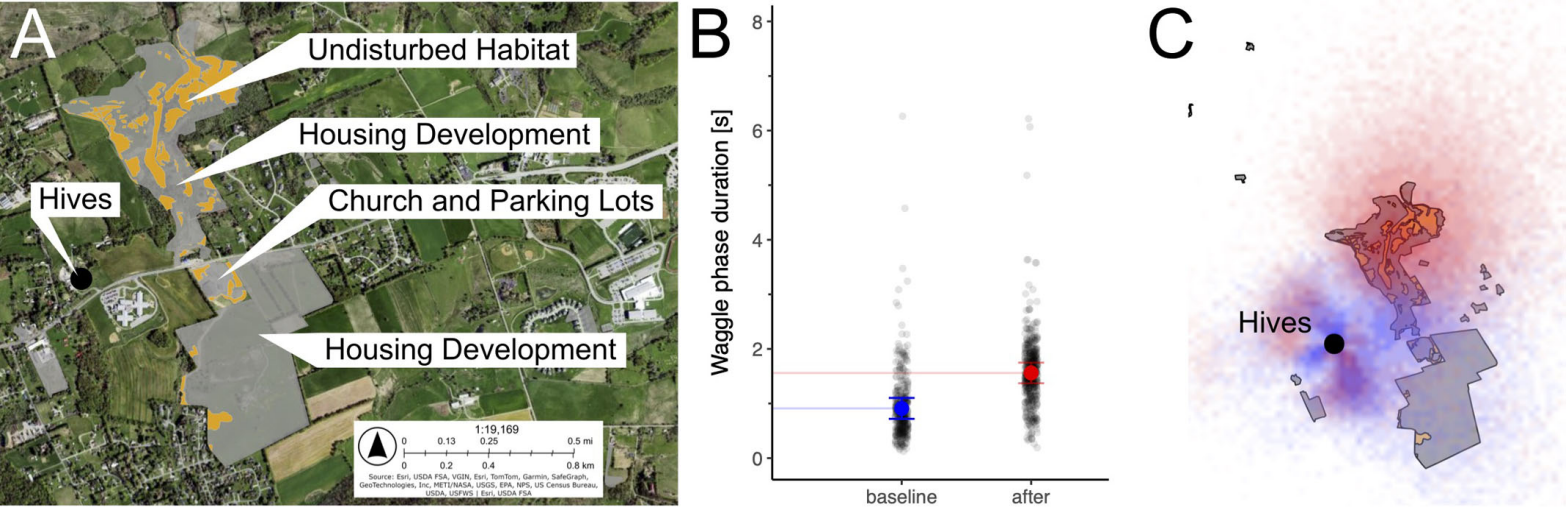
Honey bees nearly double their foraging distance by shifting and consolidating their preferred sites to remaining, isolated habitat patches within the larger land use changes. (A) Aerial imagery in 2022 of the study area in Blacksburg, VA, USA. Grey polygons represent all the lands converted in 2020-2021. Orange represents small patches of undisturbed microhabitat left within the developments. Black circle denotes the location of the hives. (B) Honey bees nearly double their communicated foraging distance in 2022 (sample size *n*=502) compared to 2018-2019 (sample size *n*=382) (mean±c.i, two-tailed likelihood ratio test used for analysis). Here each datapoint is a decoded waggle dance. (C) Honey bee foraging, as determined by dance decoding before (2018-2019, blue) and after (2022, red) the land use change, demonstrated that the bees shifted recruitment to the more distant, remaining orange patches within the grey, especially in the northern corner of the new housing development.

Here, we decoded from video, mapped, and analyzed (Couvillon et al., 2014a, 2012; Schürch et al., 2019) honey bee waggle dances from nine observation hives in Blacksburg, VA, USA, to investigate natural foraging over a mixed-use landscape that experienced land conversion: these dances occurred before (August 2018 and August 2019, *n*=382 dances) and after (August 2022, *n*=502 dances) the implementation of several large construction projects in November 2019 within the bees’ foraging range (von Frisch, 1967; Seeley, 1995; Ohlinger et al., 2024a). We hypothesized *a priori* to our study that honey bees may have increased their average foraging distance due to this construction, when we observed the bare soil it was creating. This increased foraging distance, in turn, would increase energetic cost of foraging flights, time spent foraging, and mortality risk due to predation. In particular, the construction plowed and paved over areas previously indicated by dancing bees (2018-2019) as prime habitat (Ohlinger et al., 2024a) to create houses, neighborhoods with roads, a large church, and parking lots (developments: grey polygons, Fig. 1A). The conversions retained some small, isolated patches of microhabitat (orange areas within grey polygons, Fig. 1A). As previously done, we used flight distance, encoded by the waggle run duration, as a proxy for energy expenditure (Couvillon et al., 2014a; Ohlinger et al., 2024a).

## RESULTS

We found that honey bees foraging in the post-developed landscape experienced a dramatic and significant increase in foraging distance before [average waggle run duration = 0.9 s (0.7 s to 1.1 s)] versus after [average waggle run duration = 1.6 s (1.4 s to 1.7 s)] land conversion, with a mean difference of 0.6 s [(0.4 s to 9 s); likelihood ratio test: χ²=11.007; *P*<0.001; Fig. 1B]. As duration of the waggle run (the component of the waggle dance that encodes distance to indicated resource) linearly translates to distance, this corresponds (Schürch et al., 2019) to an increase in communicated distance from approximately 0.69 km to 1.28 km. In other words, honey bees needed to fly twice as far to collect food in 2022 than they did in 2018-2019. We can estimate from published calculations that the doubling corresponds to an increased individual forager energy demand from 7.0 Joules to 13.0 Joules (one way) (Hanauer-Thieser and Nachtigall, 1995). Using an average speed of 5.4 km/h from previous literature (Tosi et al., 2017), this means foragers spent an extra 6 min 33 s each way (13 min 6 s per round trip), if they flew directly from the hive to the indicated resource at a constant speed, with no breaks or diversions, each foraging flight. This also corresponds to an increased mortality of 1.965% in foragers due to predation, per flight, again assuming they travel directly, at a constant speed, and without taking into account the time spent on the resource itself (Prado et al., 2020).

**Fig. 2.**
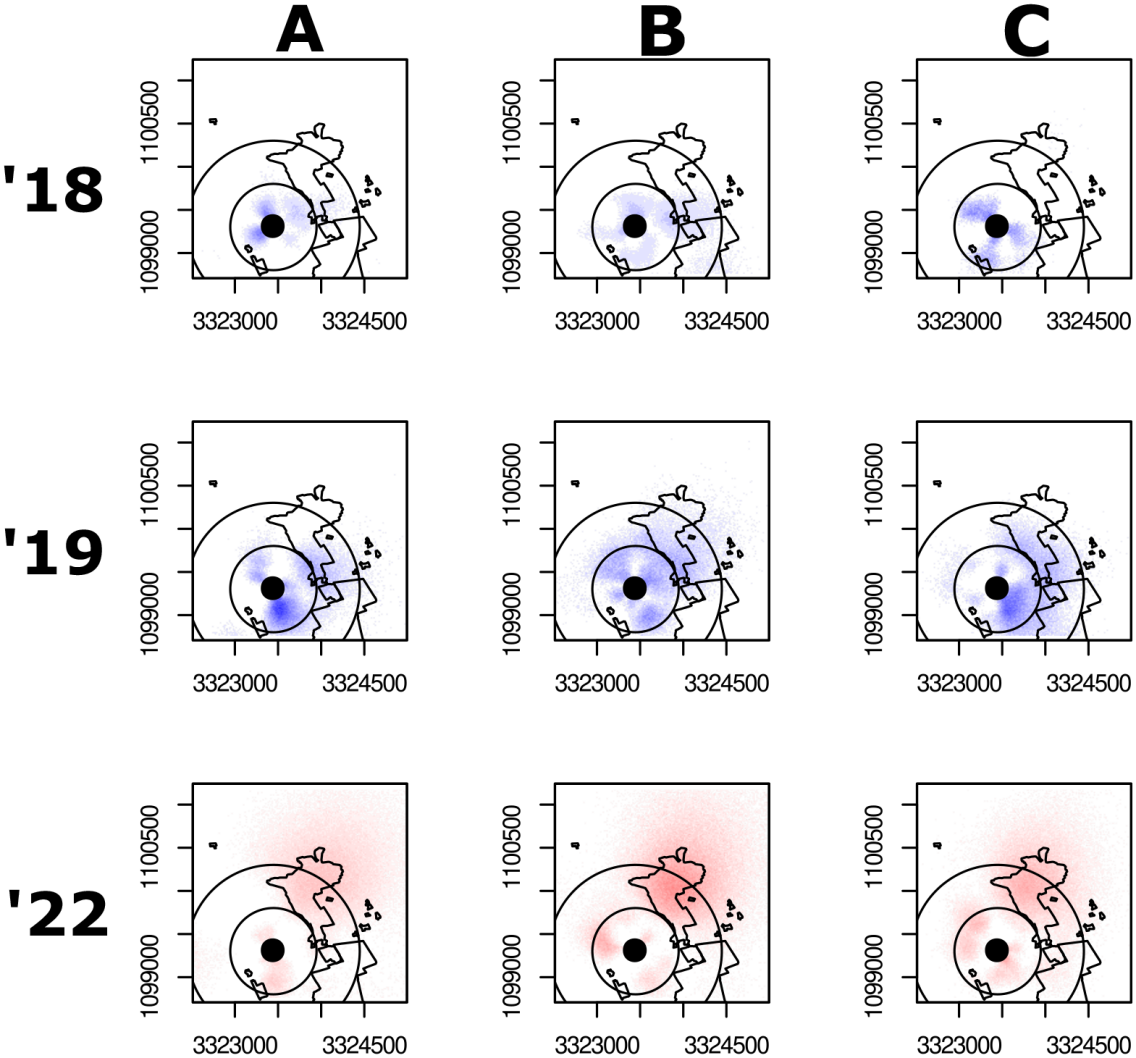
**Honey bee foraging mapped by individual hives and year.** To illustrate foraging trends were not driven by a singular, populous hive, we present each hive's foraging patterns for each year of observation. The top row represents hives from 2018, the middle row represents hives from 2019, and the bottom row represents hives from 2022. Column A shows hive A for each respective year, the middle column shows hive B for the respective years, and the right column represents the same for hive C. Note that although we labelled our hives A, B, and C each year, the observation colonies were changed each year. For example, hive A from 2018 and hive A from 2019 are different colonies, despite the shared name. We keep the red/blue colors for waggle dances as presented in Fig. 1 (blue corresponds to waggle dances prior to construction, in either 2018 or 2019, red corresponds to waggle dances following construction in 2022).

When we mapped the dances, we saw two interesting and interconnected shifts. In 2018-2019, the August bees largely advertised to two nearby, highly profitable, conjoined clusters (blue overlaid dances, Fig. 1C; Fig. 2), with the first cluster surrounding the hives, and the second, more diffuse, cluster approximately 500 m to the east and southeast in agricultural grasslands (Ohlinger et al., 2024a).

After development, the land under the second foraging cluster to the east/southeast was converted almost completely to a large church, its parking lots, and some housing developments. The resulting bare soil removed the opportunity for the bees to forage there. The bees correspondingly shifted their resource exploitation towards the back of the housing developments, about 1 km away from the hive (red overlaid dances, Fig. 1C). Particularly, the bees significantly increased their exploitation of what little small patches of habitat remained amid the cleared land: recruitment to orange areas within grey polygons increased by 3.5-fold [2018-2019=2.4% (1.0% to 3.9%); 2022=8.4% (6.2% to 10.8%); mean difference: 6.0% (3.2% to 8.8%)]. Considering how small these patches are in relation to their overall foraging range, 8.4% represents an intense usage, most likely because other options were now limited.

## DISCUSSION

As economic foragers, bees are so sensitive to energetic costs (distance) that they will only recruit their sisters as far as necessary. This means our primary outcome, communicated forage distance (decoded from waggle phase duration), can be used as a proxy for food availability (Seeley, 1995, 1994; Couvillon et al., 2014b; Garbuzov et al., 2014). Here, we see through communicated forage distance that bees are having a harder time finding profitable food after construction, needing to travel almost twice as far during foraging flights (Fig. 1B). While the near doubling of energetic costs is impressive singly, scaling to colony-level underscores the impact's extent. Approximately one third of the tens of thousands of workers are, at any point in the day, foraging (Seeley, 1995). Not only are foragers spending nearly double the energy in their foraging flights (7 to 13 Joules), they are spending over 13 min more in flight than they had to prior to construction, which leads to about 2% more foragers being predated upon than would be otherwise. The colony is now faced with less efficient foraging trips (due to the energetic cost), less total number of foraging trips (due to the increased time they take), and less total number of returning foragers (due to increased predation). These all reinforce one another, as the colony has less resources to replace foragers, raise young, and provision for winter. Ultimately, these may accumulate to a concern for the fitness of the hive as a whole (Seeley, 1995). Since foraging on these patches did not occur year-round before construction, the extent of these long-term effects is not yet fully clear.

In our study, the land developments (grey polygons), accounting for <1% of the honey bee foraging range (von Frisch, 1967), generated a considerable impact on food-collecting behaviors, nearly doubling their average foraging distance by shifting and consolidating their preferred sites to the remaining, undisturbed patches within the larger developments. It is important to remember that honey bees might actually be more resilient than other bees to land use changes because they are eusocial, capable of long-distance foraging, and utilize waggle dances (Winfree et al., 2009; von Frisch, 1967). Therefore, they can scout, discover, and recruit over large areas, allowing them to exploit remaining patches of undisturbed microhabitat, as we have seen here. Other bees from solitary nests that may only forage within tens or maximally hundreds of meters (Grüter and Hayes, 2022), or bees that depend on localized habitat for both food and housing, might experience more severe impacts. Additionally, the land conversion was only 0.9 km^2^ in total area, but it nevertheless exerted an influence on bees housed half a kilometer away. With solitary species requiring more localized floral resources, and a decreased availability in those resources for honey bees, one might predict a decrease in pollination and other ecosystem services provided by bees in and around the resulting developments. Because habitat loss decreases biodiversity, and a decrease in biodiversity decreases biomass production (which can lead back to habitat loss for bees, as they may require a certain species or amount of flora/nesting material), we can additionally anticipate synergistic consequences of land development up the trophic pyramid and throughout the ecosystem (Hooper et al., 2012).

### Next steps

We do not measure the size of, or the foraging percentage contained within the observed patterns of foraging clusters (Fig. 2), because it necessitates arbitrary decisions on where exactly they begin or end – unlike being able to measure foraging percentage within the discrete polygons drawn for construction plots and surviving microhabitat. Instead, we offer hard data on increased waggle run duration as a proxy of foraging distance. Likewise, we cannot quantitatively measure any potential increased foraging ‘spillover effect’ that remaining microhabitat may have created outside of their measured plots (that is, foraging that took place on remaining microhabitat but were mapped outside of our polygons due to bee miscommunication, decoding error, or reprojection uncertainty, and were thus excluded from analysis), because it would entail guesswork about how far away from microhabitat is appropriate to measure. Further studies may be able to develop appropriate methods for such questions, using mapped waggle dances in study locations with uniquely identifiable bees and confirmed visits to controlled and measured plots of forage.

In the past, August in Blacksburg was a time when bees could forage relatively close on average, implying that floral resources were abundant and less likely to cause scarcity/amplify the effects of habitat loss (Ohlinger et al., 2022). In previous years, bees in Price's Fork Research Center were able to forage at 500 m (Ohlinger et al., 2022). Post construction, they travelled more than 1000 m. Other months were found to have longer waggle runs (Ohlinger et al., 2022), which may suggest average distance to forage at these times may be less impacted by this construction. If they are already traveling far, removing habitat nearby may have less of an effect at that point in time.

Future studies may be conducted using these methods to determine whether habitat loss during times of less abundant floral resources have different results, and repeated use of these methods may even yield a model for to determine cost to honey bees (in time spent foraging, predation, energy expenditure, etc.) given the proximity and size of habitat loss along with temporal floral availability. Follow up studies in the same location would also be interesting, to see if attractive plants brought in from homeowners change foraging behavior.

## MATERIALS AND METHODS

### Observation hives

Using a methodology developed by our team (Couvillon et al., 2014a, 2012; Schürch et al., 2019; Ohlinger et al., 2024a), we recorded, decoded, and mapped the waggle dances of female, foraging-aged honey bees (*Apis mellifera* Linnaeus) in August of 2018, 2019 and 2022. Three colonies were studied each year in 2018-19 (six in total) as part of another project (Ohlinger et al., 2024a, 2022), and three more were studied in 2022. Importantly, sample size is number of waggle dances decoded (382 before construction and 502 after), not colony number. Further, the amount of between-bee variation is so high that any between-hive variation is negligible (Schürch et al., 2013, 2016, 2019; Couvillon et al., 2012; Schürch and Couvillon, 2014). All colonies were housed in glass-walled observation hives in our bee lab, a building at Prices Fork Research Center, Blacksburg, VA, USA (37.21148° N, 80.48935° W). We installed plumb lines, which were used as a vertical reference for dance decoding, consisting of fishing line weighted at the bottom for vertical alignment. The plumb lines were hung 5 cm apart horizontally and extended vertically down the hive. Each observation hive consisted of three full depth frames, placed vertically on top of one another, with a PVC tube (5×30 cm) connecting the hive through the building wall to the outside entrance. This allowed honey bees to forage freely in the landscape. Distinctly colored shapes were painted on the exterior wall of the building near each hive tube to minimize bee drift to different hives. Throughout the project, we practiced standard beekeeping to prevent swarming and to maintain a consistent population size across colonies.

### Recording and decoding dances

For both the previous project, which provided the August 2018-2019 data, and again in August 2022, we video recorded waggle dances from all colonies simultaneously for 1 h per day between 10:00-11:00 h EST, three to five times a week (weather permitting) at 30 fps using a Canon Vixia HF R82. We focused our camera on an area of the bottom frame, approximately 25 cm by 20 cm, where most of the dances occurred (Seeley, 1995). We saved videos to SD cards and then uploaded to Google Team Drive (GTD) for dance decoding.

Dances from 2018-2019 were already decoded as part of another project (Ohlinger et al., 2024a, 2022), with published waggle dance datasets (Ohlinger et al., 2024b). We decoded August 2022 dances using a modified methodology developed by our team (Couvillon et al., 2012) and used in previously published studies (Couvillon et al., 2014a,b; Ohlinger et al., 2022, 2024a; Silliman et al., 2022; Steele et al., 2022). Briefly, we imported videos into ImageJ (version 1.52i). We determined the angle off-set at the start of each video and then played the video until we saw the first dancing bee, which usually occurred within a few minutes. We worked through the video by decoding cohorts of simultaneous dancers. To decrease the likelihood of resampling from the same dance, we skipped ahead 6 min in the video after each decoded cohort (Couvillon et al., 2014a; Ohlinger et al., 2024a; Silliman et al., 2022; Steele et al., 2022). For each dance, we extracted the waggle phase duration, which encodes the distance to the food, and the angle relative to vertical, which encodes the direction to the food (von Frisch, 1967; Couvillon et al., 2012; Couvillon, 2012). This was done on a subset of four non-first, non-last waggle runs per dance, which has been shown to provide information consistent with averages from entire dances (Couvillon et al., 2012). Then we averaged these four runs to obtain a single duration and angle per dance (Schürch et al., 2019, 2013). As done previously, we treat each dance as an independent sample (Couvillon et al., 2014a,b; Ohlinger et al., 2022, 2024a; Silliman et al., 2022; Steele et al., 2022). In all, we decoded 502 waggle dances from August 2022. The previously published dataset provided 382 dances from August 2018 and August 2019 (Ohlinger et al., 2024b).

### Study location and time

Blacksburg is a mixed landscape, and within our honey bee foraging range (Ohlinger et al., 2024a), a circle with a radius of 3.25 kilometers, our study site prior to construction was comprised of croplands, developed lands, agricultural grasslands, and forests. A foraging radius of 3.25 kilometers was chosen because over 99% of waggle dances from 2018-2019 occurred within this zone (Ohlinger et al., 2024a). Land development and construction, notably a large church, parking lots, several neighborhoods with roads, and houses, began in November 2019 and was mostly completed by November 2022. We obtained satellite imagery from June and October of 2022, which were the nearest temporally to our video recording in August 2022. Using ArcGIS, we outlined developed land (bare soil or paved land) within a 3.25 kilometers radius of our observation hives (grey polygons, Fig. 1A) in an area that totaled 0.93 km^2^. We then created further shapes within these grey polygons to represent the remaining patches of vegetation (orange within grey, Fig. 1A). These patches totaled 0.14 km^2^. Only 1 year of post-construction data was analyzed because construction was underway in 2022, and the following year cleared flora may have regrown if it was not paved over. August of 2022 proved to be middling in terms of weather as compared to 2018 and 2019. August of 2022 had 675 accumulated degree growing days (GDD50) as compared to 2018's 657 and 2019's 681, an average temperature of 22°C compared to 2018's 21.61°C and 2019's 22.11°C, and accrued 16.79 cm of rain compared to 2018's 14.35 cm and 2019's 7.24 cm (National Weather Service Administration, N.O.a.A, 2024).

### Statistical analyses

Because waggle dances are inherently imprecise, we plot advertised foraging locations as probability distributions that reflect our uncertainty about the communicated locations (Couvillon et al., 2014a,b,c; Schürch et al., 2019; Couvillon et al., 2012; Ohlinger et al., 2022, 2024a; Silliman et al., 2022; Steele et al., 2022; Schürch et al., 2013) using Monte Carlo sampling from the universal calibration dataset that performs well across different landscapes and experimental contexts (Schürch et al., 2019; Carr-Markell and Spivak, 2021). We simulated the angular component of the dance by sampling from a von Mises distribution with a concentration parameter (κ) of 24.5, which is a circular analogue to a normal distribution (Schürch et al., 2019). Each averaged dance, consisting of the four decoded waggle phases, was then simulated 1000 times. All statistical analysis was conducted in R version 4.4.0 (R Core Team, 2024). Significance was tested for using a linear mixed effect model.

AI (Chat GPT) was used in the creation of this manuscript only to suggest synonyms and alternate phrases for original writing.
